# First validation of a model-based hepatic percutaneous microwave ablation planning on a clinical dataset

**DOI:** 10.1038/s41598-023-42543-x

**Published:** 2023-10-06

**Authors:** Bruno Frackowiak, Vincent Van den Bosch, Zoi Tokoutsi, Marco Baragona, Martijn de Greef, Aaldert Elevelt, Peter Isfort

**Affiliations:** 1 Philips Research, Data Science & Digital Twin, 5656 AE Eindhoven, The Netherlands; 2 Department of Diagnostic and Interventional Radiology, University Hospital RWTH Aachen, 52074 Aachen, Germany

**Keywords:** Oncology, Engineering

## Abstract

A model-based planning tool, integrated in an imaging system, is envisioned for CT-guided percutaneous microwave ablation. This study aims to evaluate the biophysical model performance, by comparing its prediction retrospectively with the actual ablation ground truth from a clinical dataset in liver. The biophysical model uses a simplified formulation of heat deposition on the applicator and a heat sink related to vasculature to solve the bioheat equation. A performance metric is defined to assess how the planned ablation overlaps the actual ground truth. Results demonstrate superiority of this model prediction compared to manufacturer tabulated data and a significant influence of the vasculature cooling effect. Nevertheless, vasculature shortage due to branches occlusion and applicator misalignment due to registration error between scans affects the thermal prediction. With a more accurate vasculature segmentation, occlusion risk can be estimated, whereas branches can be used as liver landmarks to improve the registration accuracy. Overall, this study emphasizes the benefit of a model-based thermal ablation solution in better planning the ablation procedures. Contrast and registration protocols must be adapted to facilitate its integration into the clinical workflow.

## Introduction

Percutaneous thermal ablation is an established minimally invasive treatment option^1–5^ for patients with primary and secondary liver tumors (i.e. hepatocellular carcinoma (HCC) or liver metastases) who are amenable for locoregional treatment but not candidates for surgery^6^. Tumor ablation relies on heat deposition from a needle shaped applicator in the target volume. Thereby, tissue necrosis is achieved when the heating temperature induces protein denaturation^7,8^. A successful thermal ablation relies both on a correct placement of the applicator and on appropriate ablation settings (i.e. power and duration of microwave application) to completely treat the lesion including a sufficient safety margin (between 5 and 10 mm^9^). Required settings are determined by the lesion size, manufacturer information (typically derived from ex vivo data) and operator experience. Vulnerable structures (e.g. bile ducts and bowel) in the vicinity of the applicator should be considered in the ablation procedure, by ensuring that their temperature stays below a safety limit. This often results in a complex out-of-plane puncture path to safely perform the procedure. Moreover, large vessels can reduce the efficacy of the thermal ablation by means of a flow mediated heat sink around the applicator^10,11^. Irregularly shaped tumors further demand thorough three-dimensional correlation between needle and tumor location, thus making the procedure more cumbersome. Therefore, there is a strong need for image guidance and feedback on the success of the procedure. Model-based thermal ablation planning is a promising approach to assist the radiologist with the procedure, providing essential information on treatment settings. This has been shown in several studies for cryoablation, radiofrequency (RF), microwave (MW) and laser ablation in (pre-)clinical scenarios^11–18^. In this study, a biophysical model of thermal ablation is integrated in a prototype software which processes magnetic resonance imaging (MRI), or computed tomography (CT) imaging acquired during the regular clinical workflow. We retrospectively validated this software prototype for planning and post-procedural ablation confirmation of CT guided MWA for hepatic malignancies. Main purpose is to demonstrate feasibility of the model and its superiority compared to manufacturer tabulated data, while identifying possible weaknesses and ways to mitigate them.

## Results

**Figure 1:**
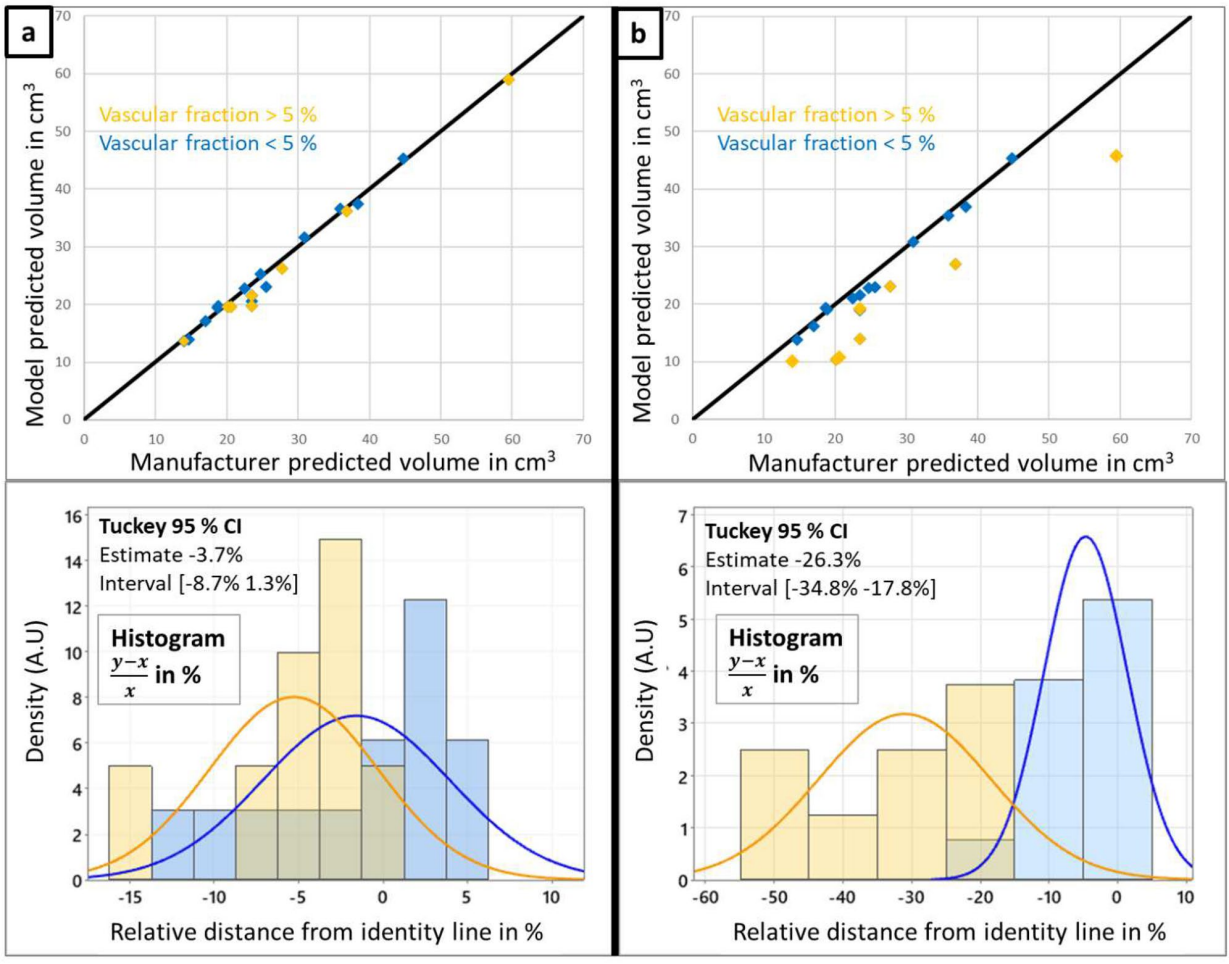
Comparison between manufacturer and biophysical model prediction of the ablation volume, influence of the vascular fraction. Histogram of relative distance from the identity line $\frac{(y-x)}{x}$. Tukey $95\%$ confidence interval range test between cases with vascular fraction above $5\%$ and the rest of the cohort. Left column (**a**) shows results without vascular cooling effect incorporated in the model. Right column (**b**) shows results with vasculature cooling effect incorporated in the model.

First scatter plots of the biophysical model predicted ablation volume against manufacturer data are shown on Fig. 1, combined with histograms of the relative distance from the identity line $\frac{(y-x)}{x}$. When vascular cooling is not incorporated in the model (by disabling the perfusion sink term on the segmented vasculature (left column a), a good correlation between respective ablation volumes can be identified ($r=0.9978$), and cases with high vascular fraction do not deviate significantly from the identity line. The corresponding Tukey’s range test shows no significant difference in the distance from the identity line between the two different vascular fraction groups. When vascular cooling is incorporated in the model (right column b), there is a clear distinction in the predictions for cases with vascular fraction above $5\%$ and the rest of the cohort, leading to a decrease in the correlation coefficient ($r=0.9759$). This distinction is also clearly captured in the Tukey’s range test on the relative distance from the identity line.

**Figure 2:**
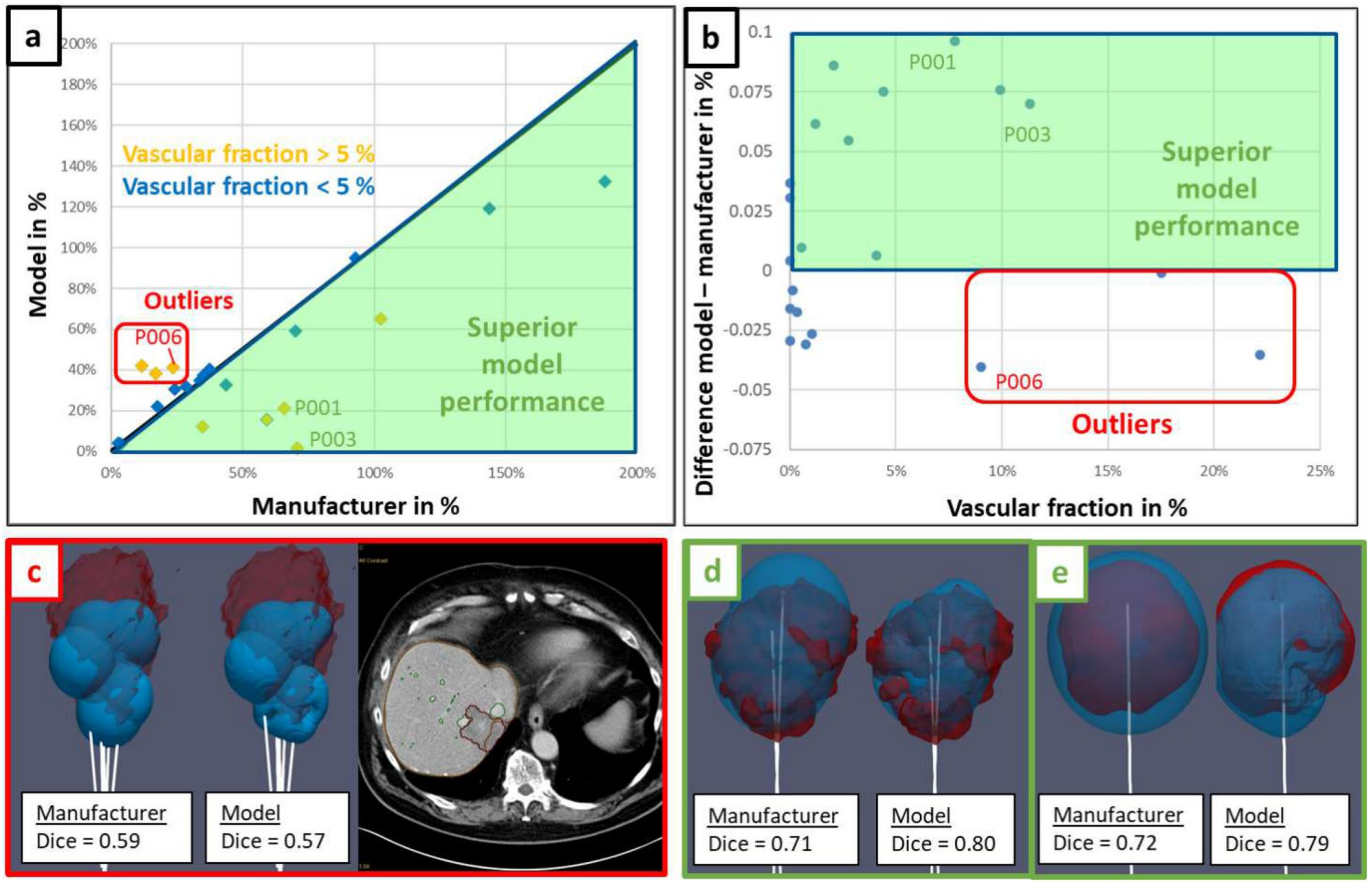
Biophysical model performance compared with manufacturer data and related to ablation ground truth. Scatter plot (**a**) corresponds to the relative ablation volume difference with ground truth $|\Delta V|/V_{GT}$. Scatter plot (**b**) corresponds to the Dice coefficient difference between model and manufacturer data as a function of the vascular fraction. Lower left corner (**c**) shows an example of outlier (patient P006) with overestimated ground truth volume and high vascular fraction. Lower right corner shows two examples of patients with superior model performance and high vascular fraction ((**d**): patient P001, (**e**): patient P003).

The relative volume difference between biophysical model prediction and ground truth ablation $|\Delta V|/V_{GT}$ is plotted against the relative volume difference between manufacturer data and ground truth ablation in Fig. 2a. In addition to those relative volumes, the difference of Dice coefficient between the biophysical model and manufacturer data is plotted against vascular fraction in Fig. 2b. Most of the points fall into the green area corresponding to a superior performance of the biophysical model. Moreover, the model performance becomes even better when the vascular fraction is large ($> 5\%$). Examples of cases with high vascular fraction (patients P001 and P003, respectively shown on Fig. 2d, e) demonstrate the better match of the model prediction to the highly distorted shape of the ground truth ablation volume. Nevertheless, three outliers can be identified on the scatter plots (red box, Fig. 2a, b), with a large vascular fraction but a lower performance of the biophysical model. As shown in the 3D visual example (patient P006, Fig. 2c), those outliers exhibit a larger ablation ground truth compared to the prediction, stretching towards the liver edge in an inconsistent manner relative to the applicator position.

**Figure 3:**
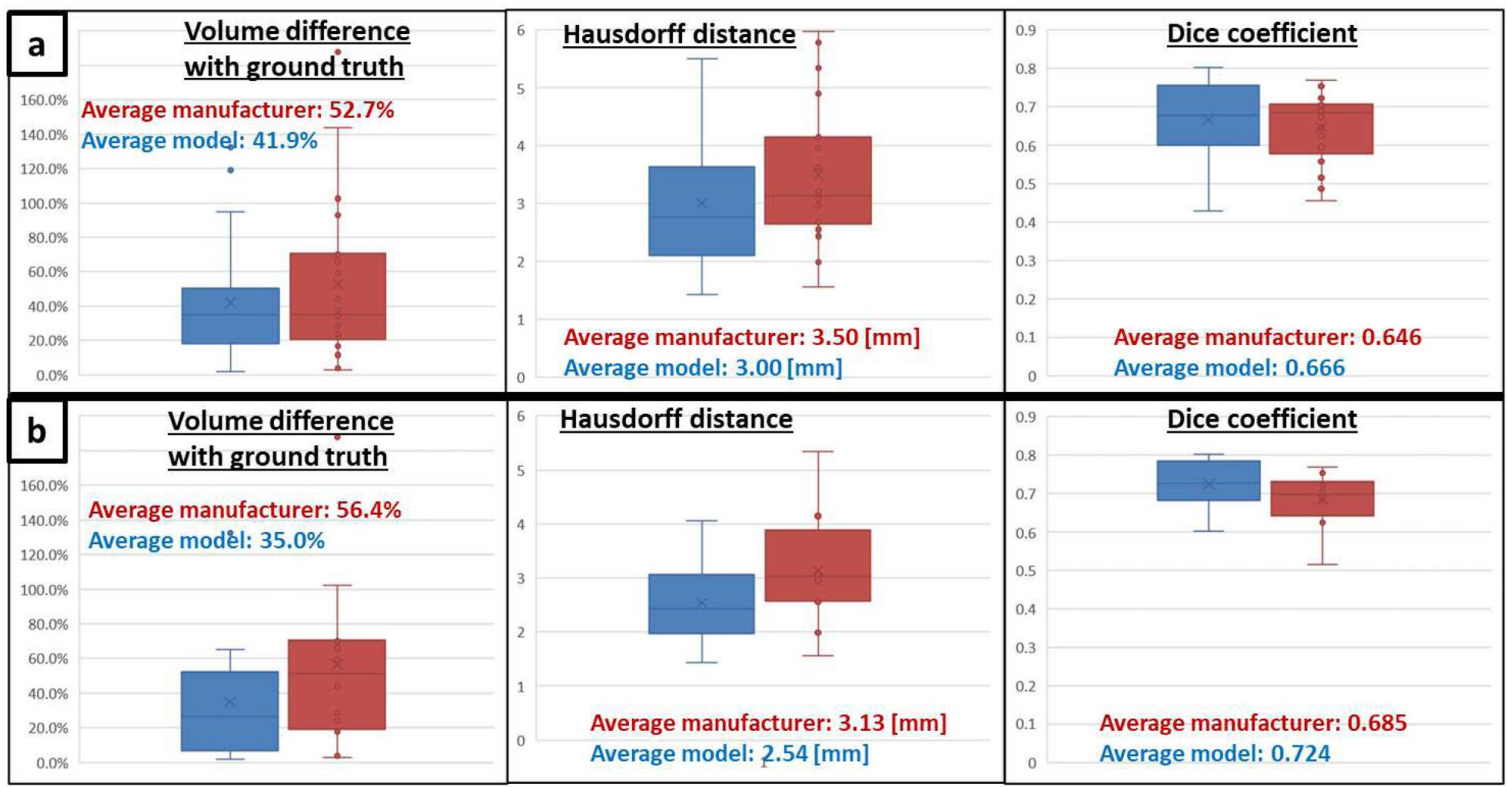
Whisker plots applied to the ablation performance indicators: volume relative difference with ground truth $|\Delta V|/V_{GT}$, absolute Hausdorff distance from ground truth and Dice coefficient. Comparison between manufacturer and model prediction. Upper row (**a**) shows results before excluding cases with overestimated ground truth volume and cases with apparent misaligned applicator (with respect to the ground truth). Lower row (**b**) shows results after applying those exclusion criteria on the cohort.

Finally, a clear superiority of the biophysical model arises from the statistical analysis performed at the aggregated data level (Whisker plots on Fig. 3) for three performance indicators: relative ablation volume difference with ground truth $|\Delta V|/V_{GT}$, absolute Hausdorff distance and Dice coefficient. By excluding cases with overestimated ground truth volume (as shown in patient P006, Fig. 2c) and cases with apparent misaligned applicator (with respect to the ground truth), the superiority of the biophysical model becomes more clear: ablation volume difference with ground truth $21.4\%$ lower, absolute Hausdorff distance $0.59$ mm lower and Dice coefficient $0.039$ higher (Fig. 3b).

## Discussion

In this analysis, the superiority of the model-based solution for predicting the thermal ablation volume has been clearly demonstrated compared to manufacturer tabulated data (Figs. 2 and 3), emphasizing the significant influence of the vasculature cooling effect for cases with vascular fraction larger than $5\%$ (threshold determined from Tuckey’s range test on the predicted ablation volume, Fig. 1). Moreover, the good correlation on the predicted volumes when the vasculature cooling effect is not incorporated in the model (Fig. 1c) demonstrates that the biophysical model is accurately matching the ablations predicted by the manufacturer in in-vivo conditions when there are no large vessels in the vicinity of the ablation. As shown on the well performing cases with strong vasculature effect (patients P001 and P003 respectively in Fig. 2d, e), cooling from larger vessels around the applicator limits MW heating which results in highly distorted ablation volumes. To the best of our knowledge, no clinical validation studies of a MWA model with perfusion cooling effect has been reported in literature. Although MWA is known to be less influenced by perfusion cooling effect compared to RFA, the ablation volumes shown on Fig. 2d, e exhibit similar distorted shapes as in the RFA guardian study^11^. This study includes perfusion cooling effect prescribed in liver tissue and on segmented vessels in a similar way as in our biophysical model. Performance metrics at the aggregated data level (Fig. 3) compares very well with reported values from validation studies in literature^11,13,15^ using RFA or cryoablation: Dice coefficient close to $0.7$ and absolute Hausdorff distance ranging between $2.3$ and $3$ mm.

Nevertheless, applicator misalignment and ground truth volume overestimation have been identified as major artifacts in the retrospective analysis, as shown by the exclusion criteria in Fig. 3. Applicator misalignment with respect to ablation ground truth affects the prediction of the manufacturer data and the biophysical model in the same way, thus resulting in comparable but poor Dice coefficients ($<0.60$). Hence, those cases do not appear as clear outliers on the graphs in Fig. 2a, b. Ground truth ablation volume and vasculature are segmented from a different CE CT scan compared to the reference scan used for the applicator positioning. Therefore, the applicator alignment with respect to the ground truth depends on the performance of the automatic registration between those scans. In practice, registration errors arise from organ displacement and deformation due to breathing or patient motion, and from the ablation itself. For the latter one, mechanical tissue displacement can be caused by the applicator, whereas tissue deformation can be caused by desiccation of the ablated volume and by swelling of the surrounding tissue^19–21^. Changing the patient’s position between diagnostic and interventional CT scan leads to additional error. Otherwise, pre-ablation scans used to position the applicator often have a limited z-axis coverage to reduce the radiation dose to the patient, thus making the registration with a CT scan with higher z-axis coverage difficult.

**Figure 4:**
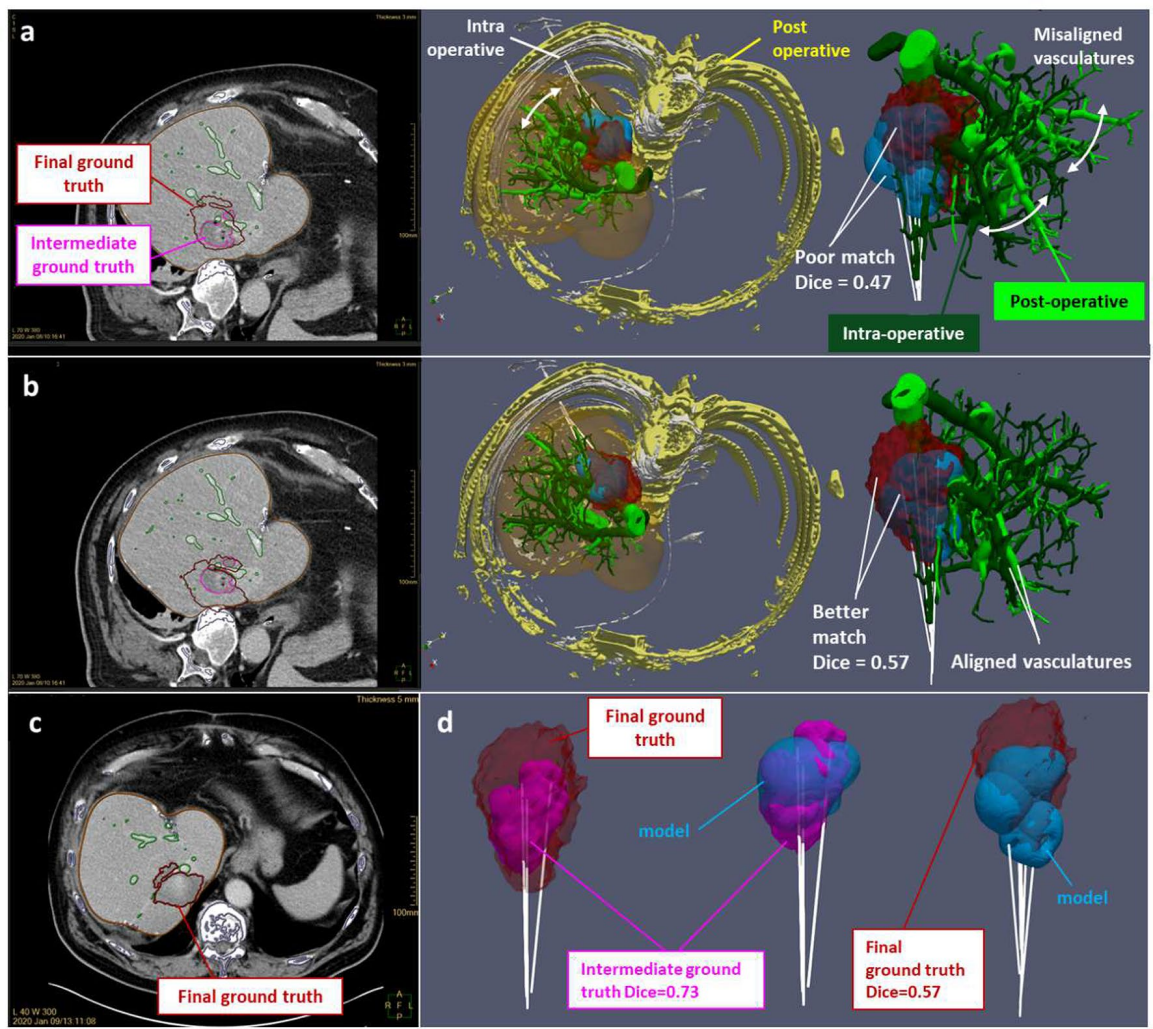
P006 patient: registration correction based on vasculature landmarks and evidence of vasculature shortage. Upper row (**a**) shows a poor match ($Dice = 0.47$) between the ablation predicted from the biophysical model and the final ground truth, and misaligned intra-operative (dark green) and post-operative (light green) vasculatures. Intermediate row (**b**) shows a better match ($Dice = 0.57$) between the ablation predicted from the biophysical model and the final ground truth, after realignment of the intra-operative (dark green) and post-operative (light green vasculatures). In the lower row, the final ground truth is shown on the postoperative scan (image (**c**)), whereas the 3D visuals (**d**) of the final ground truth (red), intermediate ground truth (pink) and biophysical model prediction (blue) evidence the vasculature shortage effect.

Registration error can be mitigated by using the vasculature as a landmark as shown on the example in Fig. 4 (patient P006). In that case, a first intra-operative CE CT scan has been performed after three ablations, leading to an intermediate ground truth matching correctly to the predicted ablation from the biophysical model ($Dice = 0.73$). However, due to an unsatisfactory treatment of the lesion, three additional ablations have been performed, and a second post-operative CE CT scan has been acquired a day after the intervention, leading to a final ground truth poorly matching to the predicted ablation ($Dice = 0.47$). Automatic registration failed in matching consistently the ablation ground truth with the applicator location as shown on the upper row a of Fig. 4. This mismatch is evidenced by comparing the respective vasculature from the post- and intra-operative CE CT scans, although the registered post-operative segmented bone shows a correct match with the intra operative one. Hence, by realigning those vasculatures (intermediate row b of Fig. 4), the match between ablation ground truth and prediction has been improved ($Dice = 0.57$). In some other cases (patients P007 and P019 shown in the Supplementary Information), vasculature landmark respective to pre- and post-ablation CE CT scans can be directly compared to evaluate a suspected applicator misalignment artefact from visual comparison between ablation ground truth and prediction.

Furthermore, ground truth overestimated volume matches better with manufacturer data on the three outliers with strong vasculature effect (Fig. 2a), because the cooling effect reduces the volume predicted by the biophysical model. This artifact evidenced on the 3D visual in Fig. 2c (patient P006) can be related to vasculature shortage^22^, where an excessive amount of heat on the vessel branch causes some local occlusion, stopping the perfusion towards the distal branches. This can lead to a temporary shutdown with consecutive liver infarction. Since both ablated liver as well as liver infarction appear as hypodense non-contrast enhancing tissue, they cannot be delineated from each other, thus creating a pronounced mismatch between predicted and ground truth ablation volume. Infarction typically stretches from the ablation zone to the liver capsule in a triangular shape. Infarction as a reason for overestimated ground truth can be proved from the evolution of the ablation ground volumes during the procedure, by comparing sequential CT control scans. Considering patient P006, the final ground truth (including all the 6 ablations, segmented from the post-operative CE CT scan 1 day after, Fig. 4c) exhibits a clear extension towards the liver edge compared to the intermediate ground truth (including three ablations, segmented from the intra operative CE CT scan, Fig. 4b). Moreover, there is a correct match ($Dice = 0.73$) between the intermediate ground truth and the predicted first three ablations from the model (Fig. 4d), meaning that the vasculature shortage is affecting the last three ablations.

Vasculature shortage remains quite complex and difficult to model, because it is influenced by the spatial distribution of the thermal dose, the vascular tree near the ablation zone and the critical ratio between local power and branch size causing occlusion (permanent or temporary). A higher probability of vasculature shortage is expected with multiple overlapping ablations, large power, proximity to the edge of the liver (because of smaller vascular branches) and a large vascular fraction^22^. Those criteria are met for patients with overestimated ground truth volume (outliers in Fig. 2a, b). For an ablation planning perspective, vasculature shortage does not matter because the predicted ablation from the biophysical model will correspond to the actual one. However, the observed ablation zone from the CE CT images after the procedure will be overestimated, with the risk that the actual ablation does not cover the lesion. Possible warning of higher vasculature shortage probability based on the above-mentioned criteria can be given to the radiologist, to increase awareness for his/her judgment on the ablation outcome, and for possibly reconsidering a control scan after a certain period if the vessel occlusion is temporary.

**Figure 5:**
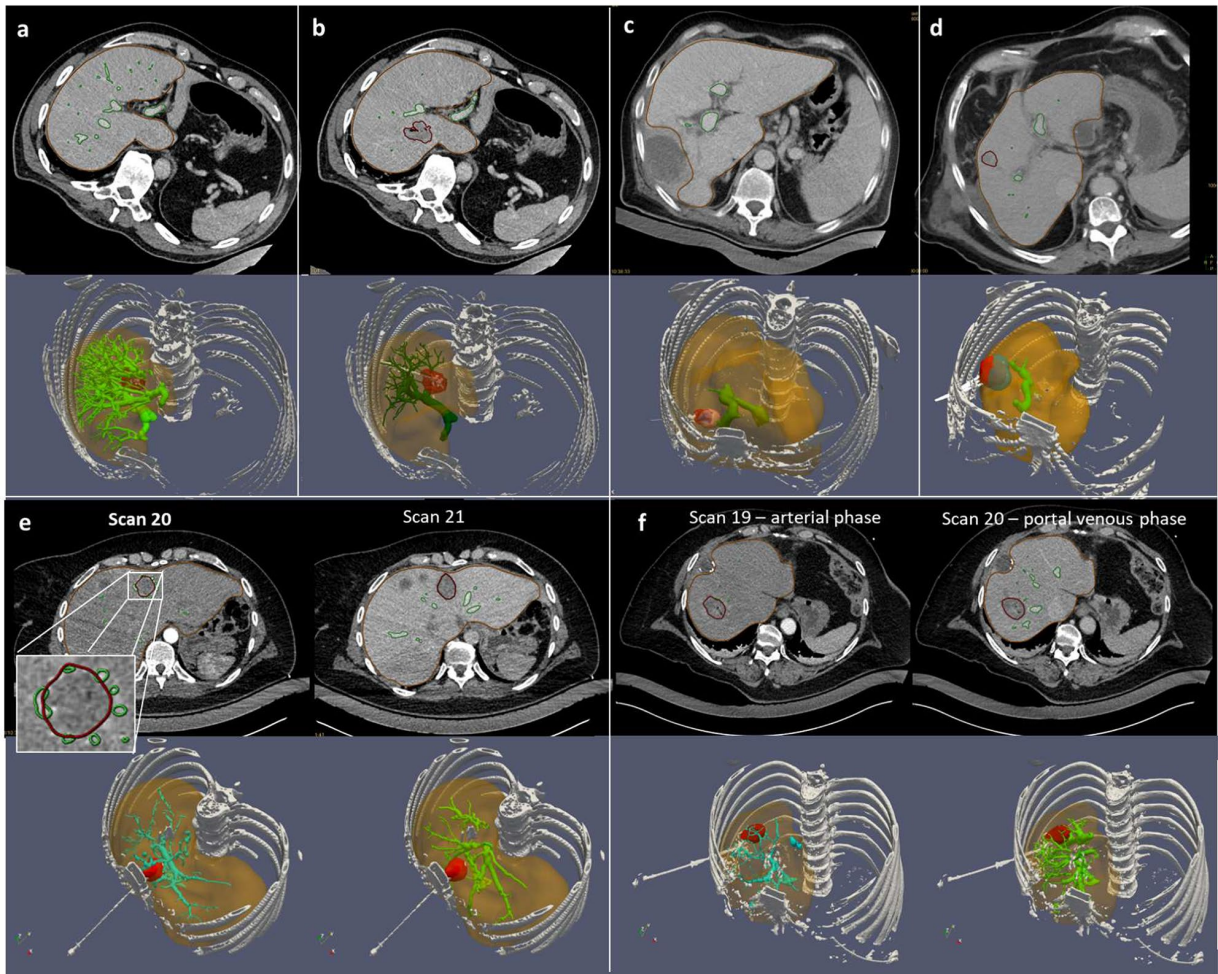
Contrast dosage and contrast phase effect on the vasculature segmentation. Upper row shows different contrast dosages for three patients ((**a**): patient P007, pre-ablation, 90 ml, (**b**): patient P007, post-ablation, 70 ml, (**c**): patient P011, 30 ml, (**d**): patient P018, 53 ml). Lower row shows different contrast phases for two patients ((**e**): patient P003, (**f**): patient P008).

Overall, the vasculature segmentation quality is a key element for the biophysical model performance, to correctly predict the perfusion cooling effect on the thermal ablation, to improve the registration accuracy using vessels as a landmark in the liver, and to assess vasculature shortage risk. It can be assessed from the minimal branch size. Branches below second level of ramification with a diameter lower than $1$ mm are not significant in terms of cooling effect^23^. Such level of vasculature quality can be achieved with standard CT imaging with thin slice reconstruction ($1$ mm). Nevertheless, the quality of the vasculature segmentation is highly related to the contrast protocol (dosage, timing) and the correct acquisition of the contrast phase. Examples a–d shown in Fig. 5 illustrate the effect of contrast dosage on the resulting vasculature, where the segmentation algorithm fails to detect the ramifications of the vascular tree on low contrast images (30 and 53 m, respectively c, d). For patient P007, the pre-ablation CE CT scan (90 ml—Fig. 5a) is preferred for the better quality of the vasculature, even if the ablation ground truth is segmented from the post-ablation CE CT scan (70 ml—Fig. 5b). Moreover, the choice of the contrast phase influences the segmentation of the vessel branches near the ablation zone. In theory, early portal venous phase is preferred because contrast enhanced blood starts to flow back from the smaller distal branches, including those around the lesion. Patient P008 shown on Fig. 5f illustrates the ideal distinction between arterial and portal venous phase in separate respective CE CT scans, as expected from the protocol. Portal venous phase in scan 20 is preferred because it captures larger vessels in the vicinity of the ablation zone, which have potentially the strongest cooling effect. However, for patient P003 shown on Fig. 5e, the distinction between arterial and venous phases is not possible on the two successive CE CT scans. A strong vascular feeding around the lesion is seen on the first scan 20, most likely related to the early portal venous phase. For this reason, despite the missing branches in the vascular tree, this first scan 20 is preferred for vasculature segmentation.

Minor changes are expected in the clinical workflow to adopt the envisioned model based thermal ablation planning solution. First, a sufficient contrast amount (minimal value of 80 ml) must be administered to allow a correct vessel segmentation. Moreover, multiple CE CT scans including the successive phases over the full sequence should be considered, with automatic merging of the respective segmented vasculatures. This will provide a complete vessel tree including the relevant branches around the lesion as an input for the biophysical model. At the end, the additional steps (e.g. segmentation, registration between scans and ablation simulation) will have a minor impact on the clinical procedure since the ablation planning tool will be integrated in the imaging system, with a biophysical model running in real time.

Our model based thermal ablation planning solution also shows some limitations. Strong tissue shrinkage has been reported and modeled for ex vivo conditions^20,21^. Nevertheless, the in-vivo behavior is more dynamic and difficult to generalize in a biophysical model. Liver tissue dielectric properties are derived from generic population data. Patient specific tissue properties could be included in the model as a next step, whereas the effect of a former ablation could be accounted in through modified tissue properties. This would pave the way for further personalization of the biophysical model and uncertainty quantification through probabilistic models^24^.

## Conclusion

A model based MWA planning solution has been validated through a retrospective analysis from a clinical dataset. Tissue perfusion has a significant impact on the quality of the ablation volume prediction. Therefore, the model needs accurate patient specific vasculature as an input, segmented from the pre/post ablation CE CT scans. In its current form, model outperforms manufacturer tabulated data. However, its accuracy can be affected by registration error and vascular effects such as local occlusion. Despite these limitations, the results show that a precise planning of ablation procedures demands software solutions such as the proposed model-based planning tool to improve ablation results and reduce the complication risks.

## Methods

### Biophysical model description

The biophysical model considers Medtronic Emprint^®^ applicator^25,26^ and predicts the time varying temperature field during the ablation procedure by solving the bioheat equation (1)from Pennes et al.^27^, where $\rho$, $C_p$, $\lambda$ and *T* are respectively the tissue density, specific heat, thermal conductivity, and temperature, whrereas the index *b* relates to the perfused blood in liver.

1 $$\begin{aligned} \rho C_p \frac{\partial T}{\partial t} = \nabla . \lambda \nabla T+\omega _{b} \rho _{b} C_{p_b}(T_{b}-T)+Q_{appl} \end{aligned}$$

The heat source term ${Q_{appl}}$ that is generated by the ablation applicator is modeled with a simplified formulation. It considers the type of the MW applicator, and it is fitted to accurately reproduce manufacturer’s specifications^28^, as verified in the subsequent section. The heat source is prescribed on the applicator active zone and relates to the ablation power and treatment duration through a temperature dependent absorption rate^23^. A distributed temperature dependent perfusion rate ${\omega _{b}}$ is applied to the full liver, as defined in Valvano et al.^29^ and in Tsafnat et al.^30^. On top of this, a perfusion sink term is applied on the subdomain defined by the segmented vasculature^31^, by enhancing the distributed perfusion rate in liver by a factor of 50 to account for the lower resistance to flow in larger vessels compared to capillary vessels. The model also includes the effect of tissue internal water evaporation through an appropriate effective heat coefficient ${Cp}$ (Yang et al.^19^). Thermal ablation volume is simulated with a damage model relying on Arrhenius equations^32,33^, which accounts for the cumulative effects of high temperatures during treatment time, and provides a *thermal dose*. Sequential ablations are considered as independent and each ablation involves a single applicator which has a single antenna. Thus, the final ablation volume is estimated by uniting all the volumes related to each ablation. Tissue shrinkage, vascular infarction or swelling during ablation are not included in the model^20,21^. The predicted ablation volume is compared to the ground truth volume at the end of the procedure. Liver tissue dielectric properties are derived from generic population data, including the influence of temperature increase during procedure up to tissue desiccation^19,34–36^. Overall, the biophysical model performs within less than a minute throughput time, thus making the planning tool compatible for a real time usage during the clinical procedure. More details on the MW model can be found in a separate contribution^28^. To uphold reproducibility of the simulations we provide a more detailed description of the model and numerical solution in the Supplementary Information. Clinical data can be made available from the University Hospital RWTH Aachen upon reasonable request.

### Clinical dataset and protocols

In the preoperative workup before a MWA procedure (not older than 4 weeks), a diagnostic contrast-enhanced (CE) CT/MRI scan is performed. A typical hepatic MWA procedure and a treatment evaluation consist of the following steps:

1. An intraoperative pre-ablation CT-scan is performed to plan the puncture. Contrast agent is administered only in case of uncertainty concerning lesion size and/or location.
2. Percutaneous placement of the applicator in an iterative (step and shoot) manner is achieved using CT guidance until the planned position is reached.
3. Ablation procedure is performed as planned.
4. The applicator is partially withdrawn to assess the ablation result properly. Withdrawal is typically done slowly with track ablation (i.e. operating the probe in a special *track ablation* setting).
5. A post-procedural CE CT scan is performed after the ablation to roughly verify if the ablation zone covers the targeted lesion with an acceptable margin (technical success) and to exclude acute complications.
6. One day after the MWA, a postoperative CE CT scan is performed to estimate technical success of the treatment and to exclude complications.
7. About 1 month after the MWA, a CE multiparametric liver MRI is performed to exclude incomplete ablation

Clinical data of a cohort of 21 de-identified patients treated with MWA for hepatic malignancies were gathered, with prior informed consent from each individual patient. The experimental protocol was approved by the local Institutional Review Board (IRB) of the *University Hospital RWTH Aachen* and all methods were carried out in accordance with relevant guidelines and regulations. All procedures were performed with the Medtronic Emprint® system^25,26^. In some cases, multiple overlapping ablations were performed. Images were acquired with a Syngo CT VB20 scanner (Siemens Healthineers, Forchheim, Germany), including at least one CE CT scan upon physician’s request. More details on the procedure are provided in Table 1.

**Table 1:** Overview of the patient cohort used in the retrospective analysis.

Patient	# abl.	Power (W)	Duration (min)	Vasc. fraction (% )	Contrast agent per CE CT scan (ml)		
					Pre	Intra	Post
P001	2	100–100	8–8	7.8		80–80	
P002	1	90	10	4.1		120	
P003	1	90	5	11.3		80	
P004	2	80–80	8–5	2.7		95–95	
P005	2	80–90	6–5	2.1		95–95	
P006	6	100–80–80–80–80–80	8–5–5–5–4–5	9.0		100–100	120
P007	2	80–80	4–3	17.5		90–70	
P008	1	100	6	4.4		100	
P009	1	100	6	0.0		50–50	
P010	1	80	6	1.0		60–60–60–60	
P011	2	80–80	8–8	0.0		30	
P012	1	100	8	0.0		100	
P013	1	80	4	9.9		90–90	
P014	2	80–80	5–10	0.1		90–90	
P015	2	80–80	10–10	0.6		70–70	
P016	2	80–80	10–10	1.2	120	50–50	
P017	2	80–80	5–5	0.0	120	100–100	
P018	3	80–80–80	10–10–10	0.0		53–53–53	
P019	2	80–80	5–3	22.2		100–100	
P020	1	100	7	0.0		60	
P021	3	100–80–80	8– 5–5	0.7		80	

(# *abl.* number of ablations, *Vasc. fraction* vascular fraction metric, explained in the next paragraph.

**Figure 6:**
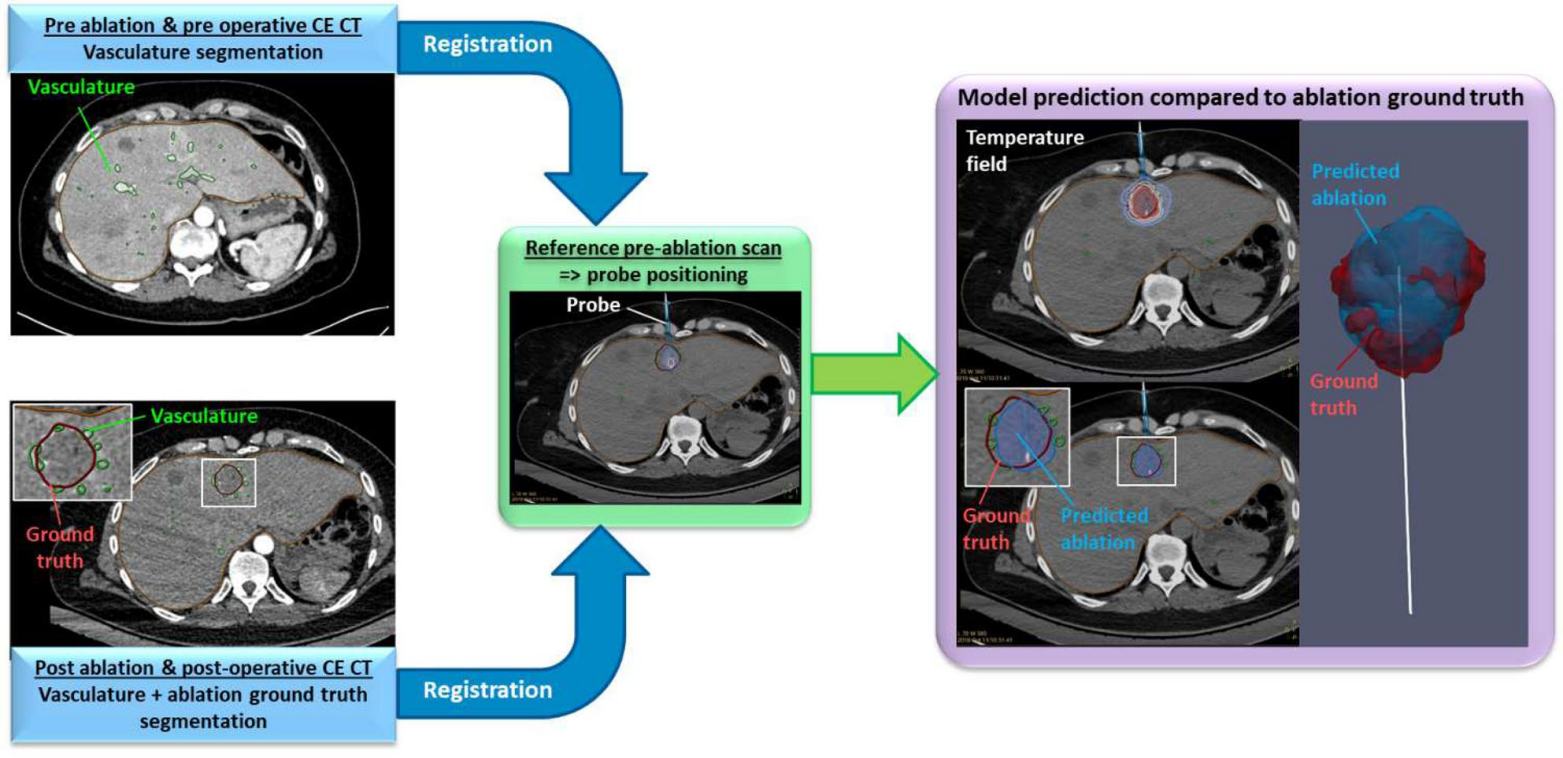
Retrospective analysis pipeline for the biophysical model validation.

The retrospective analysis workflow is shown in Fig. 6. A dedicated software combines the biophysical model with needle placement, registration, and segmentation tools from the CT/MRI images. Vasculature and ablation ground truth are segmented from the post procedural or post operative CE CT scans. The last pre-ablation CT scan is considered as the reference scan for the applicator positioning. To compensate for patient motion artefacts between CE CT scans and this reference pre-ablation scan, automatic registration is performed, accounting for translation, rotation, and elastic deformation of tissue^37^.

### Quantification of the thermal ablation performance & vasculature effect

Biophysical model performance is evaluated comparing predicted ablation volume with ground truth ablation volume. Furthermore, the Sorensen–Dice coefficient^38,39^ and the Hausdorff distance^40^ are computed. Dice coefficient (Eq. 2) quantifies the overlap between ground truth ablation volume $[A]$ and predicted ablation volume $[B]$, where 1 is a perfect match and 0 is a complete mismatch.

2 $$\begin{aligned} Dice = \frac{2|A\cap B|}{|A|+|B|} \end{aligned}$$

**Figure 7:**
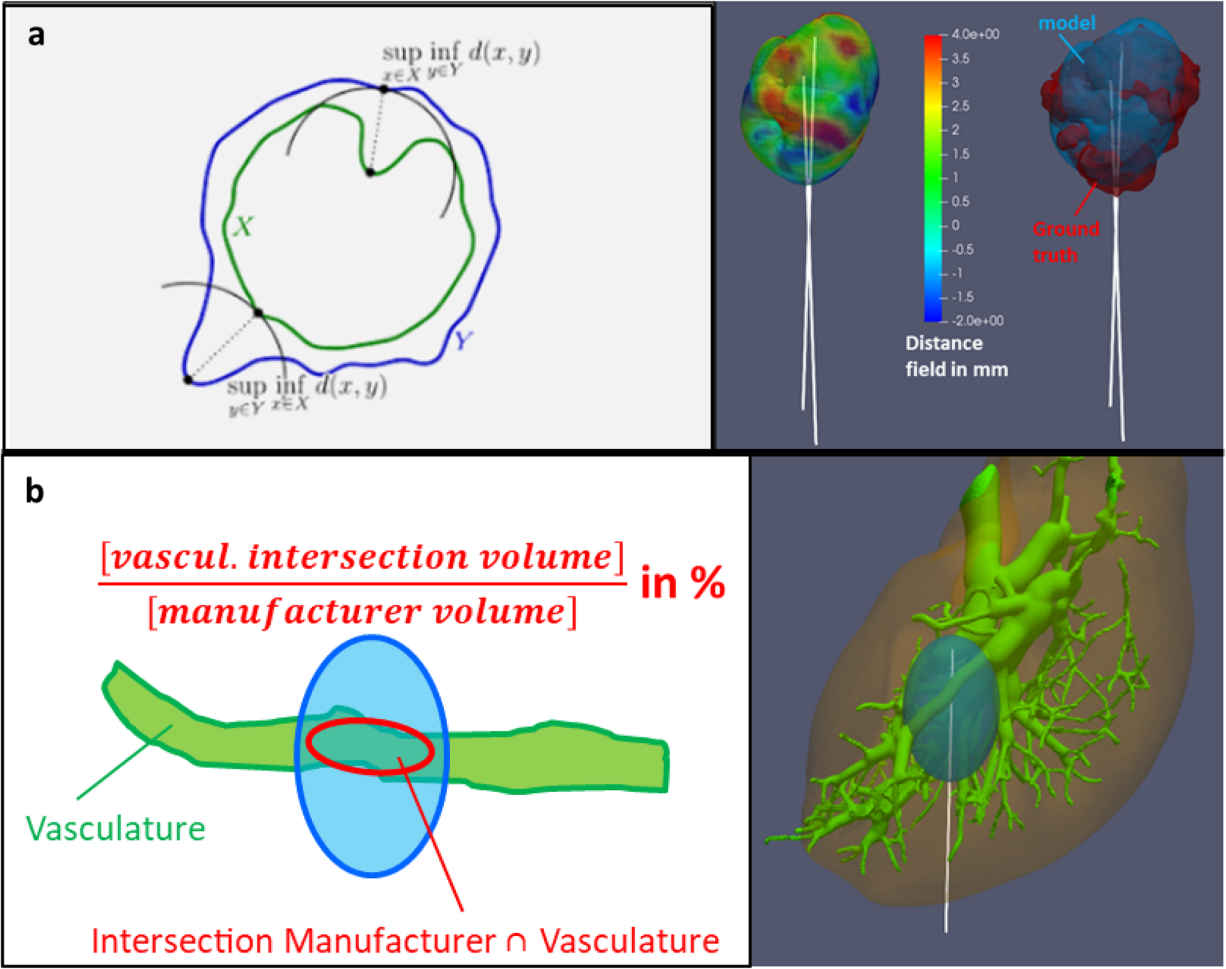
Metric to quantify the thermal ablation performance. Upper row (**a**) shows the Hausdorff distance calculation from the predicted ablation to the ground truth. Lower row (**b**) shows the vascular fraction estimation.

The Hausdorff distance from the predicted ablation to the ground truth is calculated on each point of the predicted ablation surface by considering a straight line intersecting the ground truth as close as possible, as shown in Fig. 7a. This distance can be positive, if the ground truth is enclosed within the predicted ablation volume, or negative, if the ground truth is expanding outside the predicted ablation volume.

Vasculature cooling effect is assessed prior to the simulation by quantifying the intersection between the segmented vasculature and a representative ablation volume around the applicator reported by the manufacturer, as shown on Fig. 7b. This intersecting volume is reported as a fraction of the total ablation volume, thus referred to a *vascular fraction* in the following and in Table 1.

### Reporting of the data for a statistical analysis

Statistical analysis is first performed on the ablation volume prediction (absolute value or relative difference with the ground truth), by plotting on a scatter chart the biophysical model prediction against manufacturer data. The identity line $y=x$ defines an equal prediction and the triangular area below the line clusters data with superior performance (for the difference with the ground truth). Finer clustering of data with vascular fraction is made on the absolute volume scatter plot according to a Tukey’s confidence interval range test^41^ on the relative distance from the identity line $\frac{(y-x)}{x}$. This results in a threshold value above which the vasculature effect becomes significant in shifting the points away from the identity line. Furthermore, the Dice coefficient difference between biophysical model and manufacturer data is plotted as a function of the vascular fraction: in that case the area above zero corresponds to a superior model performance. Finally, all the performance indicators are aggregated on box and whisker plots to evaluate the biophysical model performance against manufacturer data on the full cohort level. Note that the absolute Hausdorff distance is averaged over the surface of the predicted ablation zone: this corresponds to an Absolute Average Error (AAE) metric.

### Supplementary Information

Supplementary Information 1.

Supplementary Information 2.

Supplementary Information 3.

## Data Availability

The (non-personal) data that support the findings of this study are available from *University Hospital RWTH Aachen*, but restrictions apply to the availability of these data, which were used under license for the current study, and so are not publicly available. Data, with the exception of personal data, are however available from the authors associated with *University Hospital RWTH Aachen* upon reasonable request and with permission of *University Hospital RWTH Aachen* (contact person: Dr. med. Vincent Van den Bosch).

## Code availability

The final version of the code that produced the findings of this study is not publicly available since it will be implemented in a future commercial product from *Philips*. Nevertheless, a description of the code related to the biophysical model equations can be provided upon request in the Supplementary Material.
