# Author Correction: First validation of a model-based hepatic percutaneous microwave ablation planning on a clinical dataset

**DOI:** 10.1038/s41598-023-45924-4

**Published:** 2023-11-02

**Authors:** Bruno Frackowiak, Vincent Van den Bosch, Zoi Tokoutsi, Marco Baragona, Martijn de Greef, Aaldert Elevelt, Peter Isfort

**Affiliations:** 1 Philips Research, Data Science & Digital Twin, 5656 AE Eindhoven, The Netherlands; 2 Department of Diagnostic and Interventional Radiology, University Hospital RWTH Aachen, 52074 Aachen, Germany

Correction to: *Scientific Reports* 10.1038/s41598-023-42543-x, published online 06 October 2023

In the original version of this Article, Bruno Frackowiak and Vincent Van den Bosch were omitted as equally contributing authors.

The original Article has been corrected.

