# Supplementary material for: First validation of a model-based hepatic percutaneous microwave ablation planning on a clinical dataset
Source: Sci Rep. 2023 Oct 6;13:16862. doi: 10.1038/s41598-023-42543-x (PMC10558472; doi:10.1038/s41598-023-42543-x)
Supplement: Supplementary file 1 — Supplementary Information 1. [file 41598_2023_42543_MOESM1_ESM.pdf]

First validation of a model-based hepatic percutaneous microwave ablation planning on a clinical dataset (Supplementary information)

Bruno Frackowiak[1,*], Vincent Van den Bosch[2], Zoi Tokoutsi[1], Marco Baragona[1], Martijn de Greef[1], Aaldert Elevelt[1], and Peter Isfort[2]

[1]Philips Research, Data Science & Digital Twin,Eindhoven,5656AE, Netherlands
[2]Department of Diagnostic and Interventional Radiology, University Hospital RWTH Aachen, Aachen, 52074, Germany
[*]Bruno.Frackowiak@philips.com, bruno_frackowiak@hotmail.com

Microwave ablation model for Emprint devices

The model for microwave ablation used in this work is described in Tokoutsi et al.[1]. In this supplementary information, we provide further clarification to ensure the reproducibility of the results presented in this work. The design for the Emprint® applicator is intended to reproduce the Medtronic construction[2,3]. It includes the following components: a proximal choke antenna radiating element (which is connected through the inner feed to a tapered distal radiating section) and a cooling element surrounding the radiating section and acting as a buffer towards the tissue. Since the exact internal construction of the antenna is unknown, we tuned the assumed antenna design to reproduce the ablations reported by the manufacturer. This reverse engineering approach relies on the following tasks:

- Optimization of the wave reflection coefficient, as it is commonly done for MW antennas[4,5]
- Compaison between the predicted ablation and the manufacturer tabulated data under the constraint of an expected power (by also considering the efficiency that can be expected from such interstitial antennas)

This provides an effective model that reproduces very well the manufacturer data sheets, although it does not necessarily capture the exact construction of the Medtronic applicator. The Specific Absorption Rate (SAR) is simplified by decoupling the thermal effect from the electromagnetic effect, according to the following equation 1, where ε and σ are respectively the tissue electrical permittivity and conductivity, depending on the spatial coordinates x, y, z, and on the temperature T.

$$SAR(x,y,z,T) = SAR(x,y,z,T_0)f(\varepsilon(x,y,z,T),\sigma(x,y,z,T)) \tag{1}$$

This approach allows to effectively split the SAR into two parts. The first part $SAR(x,y,z,T_0)$ mainly relates to the characteristics of antenna construction and the operating frequency, T_0 beeing the initial body temperature. The second part $f(\varepsilon(x,y,z,T),\sigma(x,y,z,T))$ effectively models the change in SAR during ablation due to non-linear effects. For the Medtronic Emprint® applicator, this part is further simplified into $f = p\frac{\sigma(x,y,z,T)}{\sigma(x,y,z,T_0)}$, by dropping the dependance on the tissue permittivity ε and by using instead a scaling factor p related to the input power, as described in Table S1.

From the SAR, the deposited heat source Q_{appl} can be determined according to equation 2, where ρ is the tissue density. Thus, this heat source can be scaled with respect to its value at the initial body temperature T_0 in the same way as for the SAR. In Figure S1, we include a visual representation of this heat source $Q_{appl0} = \rho SAR(x,y,z,T_0)$ specific to the Medtronic Emprint applicator (single antenna), which is made available in the form of an unstructured visualization toolkit file (.vtu) and can be found in the additional information of this publication.

$$Q_{appl} = \rho SAR \tag{2}$$

In Table S1, we provide specifications for the tissue properties that are included in the models of the MW antenna of the Medtronic Emprint applicator[2,3], the SAR and the bioheat equation from Pennes et al.[6].

The numerical solution of the resulting Partial Differential Equations (PDEs) is achieved by utilizing an in-house implementation of Finite Element (FE) approximations of the PDEs and iterative open source solvers (relying on conjugate gradient

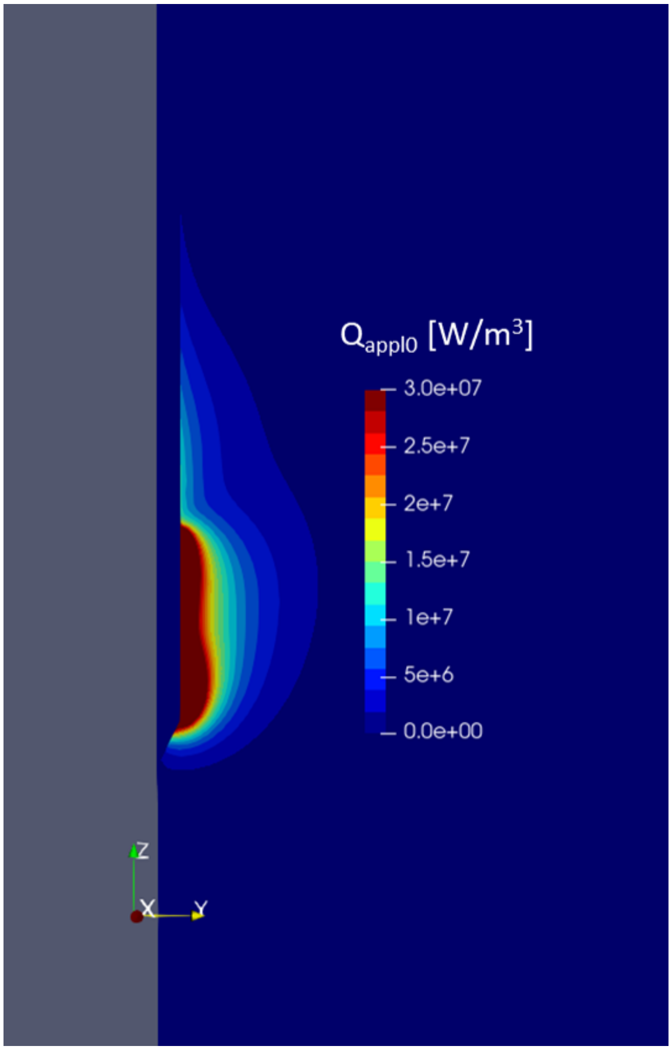

Figure S1. Visual representation of the heat source $Q_{appl0} = \rho SAR(x, T_0)$ on an axis symmetric domain generated by the Medtronic Emprint applicator[2,3] (single antenna).

methods). In particular, for both matrix assembly and solution of the discretized linear system of equations, we rely on the Eigen library[7]. The matrix assembly exploits Eigen's vectorization capabilities, following the findings described in Rahman et al.[8].

Another essential aspect of the Finite Element Method (FEM) implementation is the computational mesh. The domain discretization is at the moment realized using tetrahedrons generated by the mesh generator *Tetgen*[9]. One characteristic of the mesh generator for this application is the mesh refinement in the regions of interest, i.e. regions where the highest gradients in temperature and heat source are expected and regions where the shape of specific tissues needs to be described in detail. Hence, a two stage mesh refinement has been implemented. The initial coarse mesh is first refined according to the heat source/sink distribution. Then a second refinment step is done based on the location of the tissues of interest, e.g. segmented vasculature.

The FE discretization, the resolution of the linear system of equations, and the adaptive mesh refinement were extensively benchmarked against numerical simulations using the well established commercial solver *Comsol*[10]. The in-house implementation achieves performance that is appropriate for a forward planning usage in a clinical environmment. It typically computes a full simulation in less than 1 minute on a laptop with an 11th Gen Intel(R), Core(TM), i7-11850H, 2.50GHz and 32 GB RAM. This is much faster than typical computation times of generalist commercial software like *Comsol*[10].

Property	Details
Perfusion	As in[11,12], enhancement factor of 50 for the large vasculature
Electrical conductivity σ [S/m]	$\sigma(x,y,z,T) = \sigma_0 \left(1 - \frac{1}{1+e^{0.0697(85.375-T(x,y,z))}}\right)$ $\sigma_0 = 2.0$ [S/m]
Effective specific heat $C_{p,eff}$ [J/kg/K], W_a is the (initial) tissue water content, $W_a = W/\rho$ [-]	$C_{p,eff} = C_p - \frac{\alpha}{\rho}\frac{\partial W}{\partial T}$ $C_p = 3540$ [J/(kg K)], $\alpha = 2.26e6$ [J/kg], $W_a = 0.778$ [-] $C_{p,eff} = C_p + \alpha W_a w(T)$ $w(T[°C]) = \begin{cases} 0.2924e^{(T-106)/3.42}, T < 104°C \\ 0.0291e^{(80-T)/34.37}, T \geq 104°C \end{cases}$
SAR [W/kg] $p = 2P_{in}/100$ [-] is a scaling factor related to the power in vivo P_{in} [W] is the input power	$SAR(x,y,z,T) = SAR(x,y,z,T_0)p\frac{\sigma(x,y,z,T)}{\sigma(x,y,z,T_0)}$
Deposited Heat source [W/m^3]	$Q_{appl} = \rho SAR$
Thermal conductivity [W/m K]	$k = 0.6$ (assumed to be constant)

Table S1. Liver tissue properties and characteristics of the MW model.

Perfusion rate in liver

A distributed temperature dependent volumetric perfusion rate (expressed in $kg.m^{-3}.s^{-1}$ and applied to the full liver) is defined in Valvano et al.[11] and in Tsafnat et al.[12]. For a temperature of 37°C, this volumetric perfusion rate is equal to 5 $kg.m^{-3}.s^{-1}$. This corresponds to the product $\omega_b\rho_b$ in the bioheat equation from Pennes et al.[6], meaning that the actual perfusion rate ω_b is obtained by dividing Valvano and Tsafnat values[11,12] by the blood density ρ_b, resulting in a value of 0.0048 s^{-1}. On top of this, a perfusion sink term is applied on the subdomain defined by the segmented vasculature[13], by enhancing the distributed perfusion rate in liver by a factor of 50 to account for the lower resistance to flow in larger vessels compared to capillary vessels. This results in a volumetric perfusion rate of 250 $kg.m^{-3}.s^{-1}$ in the vasculature, thus an equivalent perfusion rate of 0.24 s^{-1}, in line with the values reported by Altrogge et al.[14]. Hence, the average perfusion rate in liver $\overline{\omega}_b$ can be determined according to the equation 3, where Vol_{liver} and Vol_{vasc} are the respective volumes of the liver and of the vasculature estimated from the segmentation tool. Table S2 provides an overview of the average perfusion rate and of the vascular fraction per patient for the considered cohort in this study.

$$\overline{\omega}_b = \frac{Vol_{liver} * \omega_b + Vol_{vasc} * \omega_b * 50}{Vol_{liver} + Vol_{vasc}} \tag{3}$$

Patient	Average perfusion rate (s^{-1})	Vascular fraction
P001	0.0128	7.8 %
P002	0.0154	4.1 %
P003	0.00712	11.3 %
P004	0.00919	2.7 %
P005	0.00919	2.1 %
P006	0.0132	9.0 %
P007	0.0139	17.5 %
P008	0.0100	4.4 %
P009	0.00968	0.0 %
P010	0.0171	1.0 %
P011	0.00686	0.0 %
P012	0.00743	0.0 %
P013	0.0110	9.9 %
P014	0.0127	0.1 %
P015	0.00919	0.6 %
P016	0.00988	1.2 %
P017	0.0121	0.0 %
P018	0.00739	0.0 %
P019	0.0150	22.2 %
P020	0.00924	0.0 %
P021	0.00933	0.7 %

Table S2. Overview of the patient cohort used in the retrospective analysis: blood perfusion in liver

Additional figures

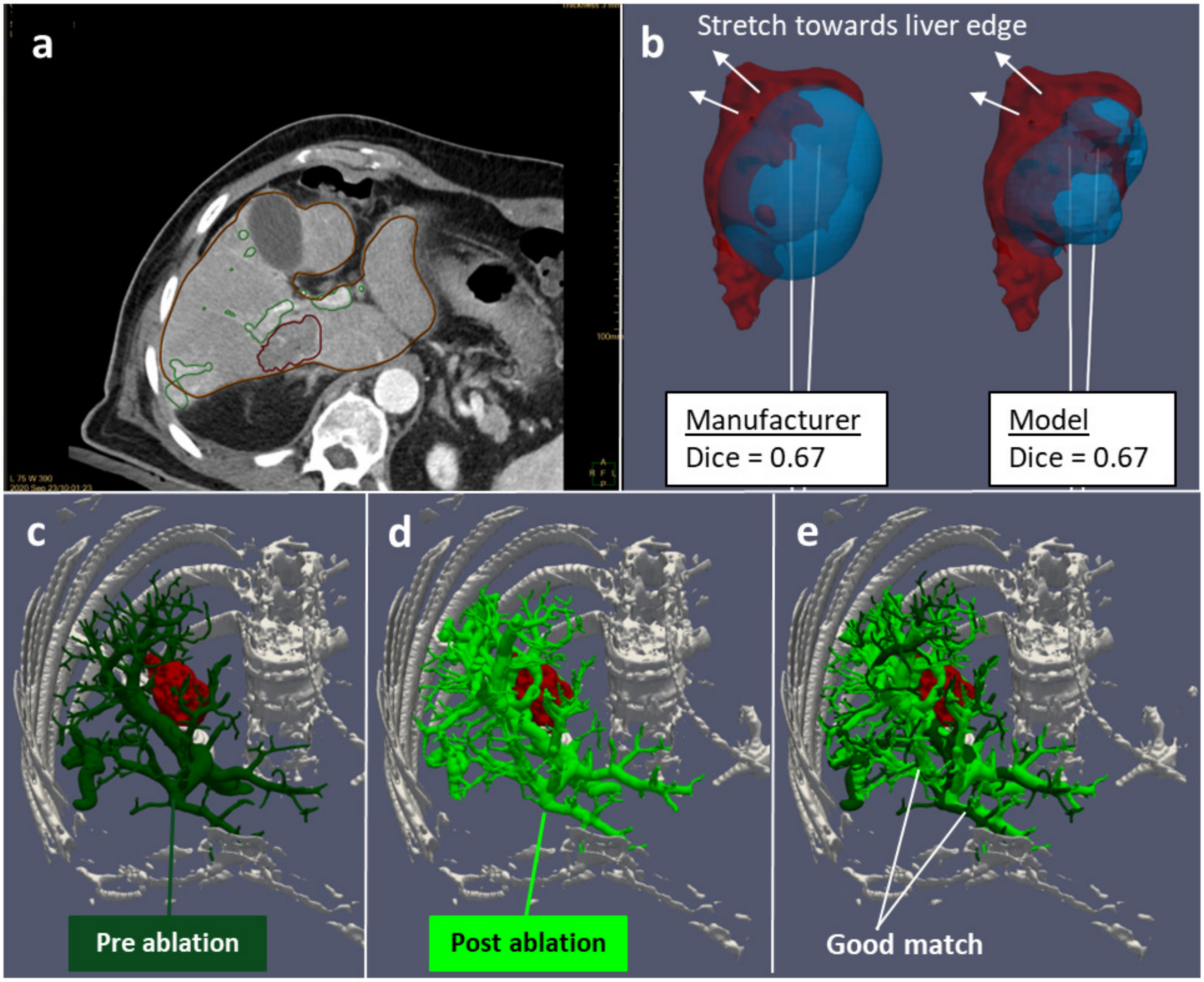

Figure S2. P019 patient. Post ablation CE CT scan **a** and 3D visuals in **b** comparing ablation ground truth (in red) with predictions from the biophysical model and from manufacturer data evidence the vasculature shortage effect, with a clear stretch of the ablation ground truth towards the liver edge. Pre- and post- ablation segmented vasculatures shown in 3D visuals in **c**, **d** and **e** demonstrate the good performance of the registration algorithm for this specific case (matching vasculatures), resulting in a correct alignment between the ablation ground truth and the applicators. Ground truth overestimation due to the vasculature shortage remains the main artifact.

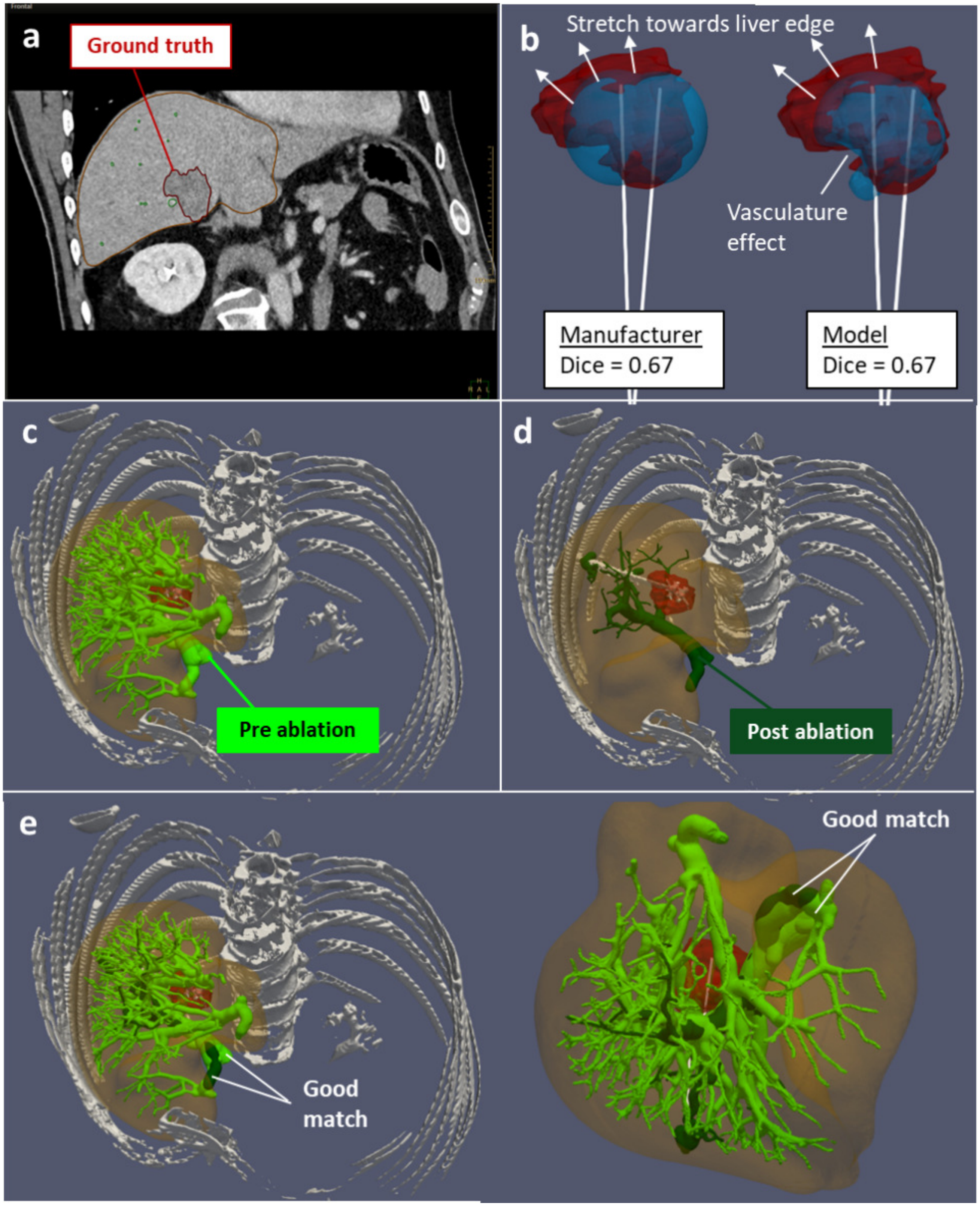

Figure S3. P007 patient. Post ablation CE CT scan **a** and 3D visuals in **b** comparing ablation ground truth (in red) with predictions from the biophysical model and from manufacturer data evidence the vasculature shortage effect, with a clear stretch of the ablation ground truth towards the liver edge. Pre- and post- ablation segmented vasculatures shown in 3D visuals in **c**, **d** and **e** demonstrate the good performance of the registration algorithm for this specific case (matching vasculatures), resulting in a correct alignment between the ablation ground truth and the applicators. Ground truth overestimation due to the vasculature shortage remains the main artifact.

References

1. Tokoutsi, Z., Baragona, M. & Frackowiak, B. Effective models of microwave antennae for ablation treatment planning. *2021 43rd Annu. Int. Conf. IEEE Eng. Medicine & Biol. Soc. (EMBC)* 4307–4310 (2021).
2. Brannan, J. Thermal ablation: understanding the breakthrough to predictability. spherical ablations with the thermosphere technology (2016).
3. Medtronic emprint ™applicator. https://www.medtronic.com/covidien/en-us/products/ablation-systems/emprint-ablation-system.html.
4. Fallahi, H. & Prakash, P. Antenna designs for microwave tissue ablation. *Critical Rev. Biomed. Eng.* **46**, 495–521 (2018).
5. Gas, P. *The S11-parameter Analysis of Multi-slot Coaxial Antenna with Periodic Slots*, 367–376 (Springer International Publishing, Cham, 2018).
6. Pennes, H. H. Analysis of tissue and arterial blood temperatures in the resting human forearm. *J. Appl. Physiol.* **85**, 5–34 (1948). http://jap.physiology.org/content/85/1/5.full.pdf.
7. Eigen library. https://gitlab.com/libeigen/eigen.
8. Rahman, T. & Valdman, J. Fast matlab assembly of fem matrices in 2d and 3d: Nodal elements. *Appl. Math. Comput.* **219**, 7151–7158, DOI: https://doi.org/10.1016/j.amc.2011.08.043 (2013). ESCO 2010 Conference in Pilsen, June 21- 25, 2010.
9. Si, H. Tetgen, a delaunay-based quality tetrahedral mesh generator. *ACM Trans. Math. Softw.* **41**, DOI: 10.1145/2629697 (2015).
10. Comsol multiphysics. https://www.comsol.com/.
11. Valvano, J. W., Cochran, J. R. & Diller, K. R. Thermal conductivity and diffusivity of biomaterials measured with self-heated thermistors. *Int. J. Thermophys.* **6**, 301–311, DOI: 10.1007/BF00522151 (1985).
12. Tsafnat, N., Tsafnat, G., Lambert, T. & Jones, S. Modelling heating of liver tumours with heterogeneous magnetic microsphere deposition. *Phys. Medicine Biol.* 2937–2953 (2005).
13. Vaidya, N., Baragona, M., Lavezzo, V., Maessen, R. & Veroy, K. Simulation study of the cooling effect of blood vessels and blood coagulation in hepatic radio-frequency ablation. *Int. J. Hyperth.* **38**, 95–104, DOI: 10.1080/02656736.2020.1866217 (2021). PMID: 33530763, https://doi.org/10.1080/02656736.2020.1866217.
14. Altrogge, I. *et al.* Sensitivity analysis for the optimization of radiofrequency ablation in the presence of material parameter uncertainty. *Int. J. for Uncertain. Quantification* 295–321 (2012).
